#  Evidence of Physiotherapy Interventions for Patients with Chronic Neck Pain: A Systematic Review of Randomised Controlled Trials

**DOI:** 10.1155/2013/567175

**Published:** 2013-04-15

**Authors:** Pia Damgaard, Else Marie Bartels, Inge Ris, Robin Christensen, Birgit Juul-Kristensen

**Affiliations:** ^1^Research Unit of Musculoskeletal Function and Physiotherapy, Institute of Sports Science and Clinical Biomechanics, University of Southern Denmark, Campusvej 55, 5230 Odense M, Denmark; ^2^Department of Rehabilitation, Aeroe Municipality, 5970 Aeroeskoebing, Denmark; ^3^The Parker Institute, Department of Rheumatology, Copenhagen University Hospital, 2000 Frederiksberg, Copenhagen, Denmark; ^4^Bergen University College, Institute of Occupational Therapy, Physiotherapy and Radiography, Department of Health Sciences, 5020 Bergen, Norway

## Abstract

Chronic neck pain (CNP) is common and costly, and the effect of physiotherapeutic interventions on the condition is unclear. We reviewed the literature for evidence of effect of physiotherapy interventions on patients with CNP. Five bibliographic databases (MEDLINE, EMBASE, CINAHL, Cochrane Library, and PEDro) were systematically searched. Randomised, placebo and active-treatment-controlled trials including physiotherapy interventions for adults with CNP were selected. Data were extracted primary outcome was pain. Risk of bias was appraised. Effect of an intervention was assessed, weighted to risk of bias. 42 trials reporting on randomised comparisons of various physiotherapy interventions and control conditions were eligible for inclusion involving 3919 patients with CNP. Out of these, 23 were unclear or at high risk of bias, and their results were considered moderate- or low-quality evidence. Nineteen were at low risk of bias, and here eight trials found effect on pain of a physiotherapy intervention. Only exercise therapy, focusing on strength and endurance training, and multimodal physiotherapy, cognitive-behavioural interventions, massage, manipulations, laser therapy, and to some extent also TNS appear to have an effect on CNP. However, sufficient evidence for application of a specific physiotherapy modality or aiming at a specific patient subgroup is not available.

## 1. Introduction

Musculoskeletal disorders are threatening quality of life by having the potential to restrict daily activities, cause absence from work, and result in a change or discontinuation in employment. These disorders are expensive for society and for patients and are responsible for the highest number of healthy years lost [1–4]. The prevalence of chronic neck pain varies. The 12-month prevalence of pain typically ranges between 30% and 50%; the 12-month prevalence of activity-limiting pain is 1.7% to 11.5% [5]. The annual incidence of neck pain associated with whiplash varies greatly. Although 50% of whiplash victims recover in three to six months, 30% to 40% have persisting mild to moderate pain and 10% to 20% retain more severe pain [6]. It is a multifaceted phenomenon with physical impairment, psychological distress, and social dysfunction, which calls for an evidence-based, cost-effective rehabilitation treatment [7–11]. 

 According to a Dutch study, 44% of patients with chronic neck pain visited their general practitioner (GP) with the condition during a twelve-month period; 51% of these were referred to physiotherapy treatment [12]. Knowledge of the actual effect of physiotherapy is therefore important and is anticipated to be reflected in the awareness of evidence-based practice among physiotherapists.

The Cochrane Collaboration has provided systematic reviews on the effect of massage for mechanical neck disorders [13], patient education for neck pain [14], electrotherapy for neck pain [15], mechanical traction for neck pain with or without radiculopathy [16], and conservative treatment for whiplash [17]. The overall conclusion has been that the evidence for these treatments is low and that no definite statements on the efficacy and clinical usefulness of these treatments can be made. A further Cochrane Review on the effect of manipulation and mobilisation of neck pain found low quality evidence that cervical and thoracic manipulations may provide pain reduction [18]. An additional Cochrane Review on the effect of exercises for mechanical neck disorders concluded that the summarised evidence indicates that there is a role for exercises in the treatment of acute and chronic mechanical neck pain plus headache but that the relative benefit of each type of exercise needs extensive research [19].

However, none of these reviews have covered the majority of commonly used physiotherapy modalities in one in order to get an overview of the subject. Besides, the effect of specific physiotherapy treatments in specific subgroups of chronic pain patients is an important topic which has not yet been examined. Clinicians and policy makers need evidence from research to inform and guide clinical practice and policy. Patients and researchers also need such information to support shared decisions and to set priorities for future research.

The aim of this study was to review the literature systematically and discuss the quality of evidence of commonly used physiotherapy interventions (exercise, manual therapy, and electrotherapy) aimed at improving outcomes (on pain, function, and quality of life) important for patients with chronic neck pain [20]. Neck pain was defined as pain located in the anatomical region of the neck [21]. Pain was considered chronic if it had persisted for more than three months, as defined by the International Association of the Study of Pain. 

## 2. Methods

We performed a systematic review of all available randomised controlled trials on the subject of physiotherapy for neck pain to determine the effects of physiotherapy interventions on pain, function, and quality of life in neck-pain patients and to explore whether beneficial effects could be explained by biases affecting individual trials [22]. Study selection, assessment of eligibility criteria, and data extraction were carried out based on a predefined, peer-reviewed protocol according to the Cochrane Collaboration's guidelines [23]. This paper was prepared in accordance with the PRISMA statement [24].

### 2.1. Literature Search

We searched five bibliographic databases (MEDLINE, EMBASE, CINAHL, Cochrane Library, and PEDro) from January 1990 to January 2012 with a structured, pre-defined, search strategy [25]. The search strategy was “Neck Pain AND Physiotherapy Intervention.” For neck pain, the following terms were combined with OR: “whiplash/WAD,” “neck injury,” “neck sprain/strain,” “neck ache,” “cervical sprain/strain,” “cervical disorder/syndrome,” “cervical spondylosis/itis,” “cervical osteoarthritis”, “cervicodynia”, “cervicobrachial pain/disorder/syndrome”, “myofascial pain/disorder/syndrome,” “trapezius myalgia,” “postural syndrome,” and “nonspecific neck pain.” For physiotherapy interventions, the following terms were combined with OR: “physiotherapy,” “physical therapy,” “rehabilitation,” “intervention studies,” “exercise,” “exercise therapy,” “exercise movement techniques,” “manual therapy,” “manipulative medicine,” “mobilisation/mobilization,” “musculoskeletal techniques,” and “electric/electro stimulation therapy.” All terms were searched as free text as well as keywords, where this was applicable. Limitations were human studies in the English, German, Dutch, Danish, Norwegian, and Swedish languages, in the time span of January 1990 to January 2012. To assure that the included studies followed scientifically sound methods and the data therefore were well documented, we set a limit for inclusion to publications from 1990 and onwards. 

Reference lists of review articles and included studies were searched to identify other potentially eligible studies. An additional search was conducted via the scientific search machine http://www.scirus.com/, using the following search terms combined with AND: “chronic neck pain,” “physiotherapy.”

### 2.2. Selection Criteria

Studies were included if participants were older than 18 years of age and had chronic neck pain for more than three months (therefore considered chronic). Chronic neck pain was defined as (i) chronic whiplash-associated disorders (WAD); (ii) chronic non-specific neck pain, including work-related neck pain, myofascial neck pain, upper trapezius myalgia, chronic neck pain associated with degenerative findings with or without radicular findings, or other surrogate terms. 

Eligible interventions were physiotherapy interventions commonly used in the treatment of musculoskeletal pain: (i) exercise therapy, including specific types of exercises, for example, neuromuscular training, strength training, and endurance training; (ii) manual therapy, for example, massage, manipulations, and mobilisations; (iii) electrotherapy, for example, TENS, low-level laser, or other surrogate terms. Acupuncture was not considered a physiotherapy technique since this technique is not part of physiotherapy in all countries. Comparison of the therapy had to be made with no treatment (e.g., waiting list controls), or other conservative active therapies called “care as usual,” or sham therapy. Anticipating that only a limited number of trials available used placebo/sham control, we decided also to include trials in which an active control was used as a cointervention. 

To be eligible for inclusion, a study must apply at least one pain measurement prior to and following the intervention, which was an outcome considered to be of major importance to the patients. Self-reported function and disability [26], self-reported quality of life [27], objective physical function, and clinical tests were considered minor outcomes and therefore not considered necessary inclusion criteria [28–30]. Only randomised controlled trials were accepted. Exclusion criteria were studies with participants with acute or subacute neck pain, neck pain with definite or possible long tract signs, neck pain due to specific pathological conditions (e.g., fractures, tumours, infections, inflammatory processes, ankylosing spondylitis, and rheumatoid arthritis), and headache.

We created a reliable process through consequently two reviewers who independently conducted the study selection and assessment of eligibility criteria. Similarly, two reviewers independently conducted data abstraction and assessed the risk of bias. Disagreements were resolved through consensus with a third reviewer being consulted if there was disagreement. 

### 2.3. Data Extraction and Evidence Synthesis

Data regarding publication status, trial design, patient characteristics, treatment regimens, outcome methods, results, and funding were extracted on a standardised form using a custom-made Microsoft Excel spreadsheet. 

We assessed the risk of bias by using the Cochrane Collaboration's tool for assessing risk of bias as presented in [23]. Each of the following domains would be considered adequate—that is, presumably with a low risk of bias (i) “adequate sequence generation”; (ii) “allocation concealment”; (iii) “blinding”; (iv) “incomplete outcome data addressed”; (v) “free of selective outcome reporting”; (vi) “free of other bias (i.e., whether a study sponsor would benefit economically from a positive outcome). Each of these key components of methodological quality was assessed on an Adequate/Unclear/Inadequate basis. We used The Cochrane Collaboration's approach for summary assessments of the risk of bias for each important outcome across domains within a trial [23].

Due to the limited number of studies investigating each of the specific interventions, it was decided that both meta-analytical and level of evidence approaches would be inappropriate. Therefore, a narrative approach where we evaluated the study and results between groups within a trial was used to summarise the findings. To formulate conclusions, only results from trials at low risk were considered as evidence for an intervention. 

## 3. Results

**Figure 1:**
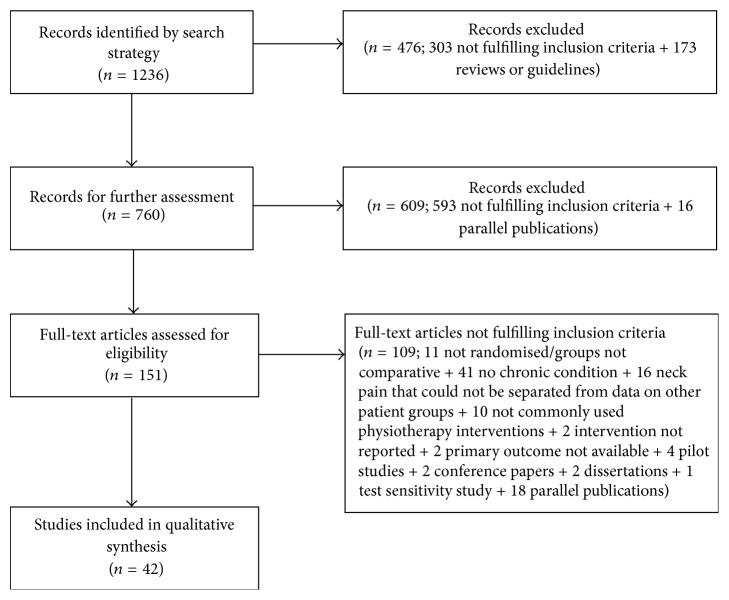
Flow diagram of the selection process of included studies.

The literature search identified 4921 relevant studies (1110 from EMBASE, 1568 from MEDLINE, 1239 from CINAHL, and 491 from PEDro), of which 3685 were duplicates, leaving 1236 potentially eligible studies to be screened (see Figure 1). Following screening of titles and abstracts, 151 potentially relevant studies were identified and retrieved in full text. Finally, 42 randomised controlled trials, involving 3919 patients, fulfilled the selection criteria and were considered suitable for inclusion. The selection process and reasons for exclusions are presented in Figure 1. 

### 3.1. Study Characteristics

Study characteristics and study results are presented under the categories exercise therapy (25 trials, 18 regarding chronic non-specific neck pain, and seven regarding chronic neck pain related to whiplash); manual therapy (six trials, all related to chronic non-specific neck pain); and electrotherapy (11 trials, all related to chronic non-specific neck pain) in Appendix A, Tables 1–4. 

The trials covered the following intervention topics: (i) exercise therapy: various types of dynamic and isometric exercises, general aerobic exercises, exercises with a focus on strength, endurance, proprioception and coordination, specific neck stabilising exercises, craniocervical-flexion exercises, posture, behavioural graded activity, relaxation, body awareness, myo-feedback training, and multimodal physiotherapy; (ii) manual therapy: massage, manipulation, and traction; (iii) electrotherapy: laser, transcutaneous nerve stimulation (TENS), ultrasound, and repetitive magnetic stimulation (rMS).

Sham therapy or waiting list controls were used as control groups in 12 trials; 10 trials used a control group consisting of a self-management book, health-counselling, or other interventions, clearly distinguished from the active intervention group; six trials used active-treatment control reported as “treatment as usual”; active-treatment control was used in 14 trials. 

Primary outcome measures were self-reported pain and/or self-reported pain and disability in 41 trials; when primary outcome measures were not reported, all outcome measures were considered. One trial had an objective test as primary outcome, yet pain was included in the secondary outcome measures.

### 3.2. Risk of Bias

Risk of bias is presented in Appendix B, Table 5.

Overall, the quality of reporting on methodological issues varied. Table 5 shows the judgements (“Adequate,” “Unclear,” and “Inadequate”) for each of the domains. As can be seen, 28 of 42 trials succeeded in reporting on adequate sequence generation; 18 trials described adequate allocation concealment; four trials adequately reported on attempts to blind participants, personnel, and outcome assessors; 22 trials adequately reported on missing outcome data, using intention-to-treat analysis; three trials adequately reported on selective outcome reporting by referring to a published and available protocol for comparisons; and 25 trials adequately reported on funding and the role of funding. 

The summary assessment of risk of bias revealed 19 trials at low risk of bias [34–39, 45, 47–49, 51, 55–60, 63, 64] and 23 trials as unclear or at high risk of bias [31–33, 40–44, 46, 50, 52–54, 61, 62, 65–72], and for this reason their results were not considered as evidence. Of these 19 trials at low risk of bias, 11 trials found no difference between intervention groups [34, 36–39, 45, 48, 49, 56, 58, 60], and eight trials found an effect on pain of the intervention [35, 47, 51, 55, 57, 59, 63, 64]. 

All studies are described in detail in Appendix A, Tables 1–4. All trials at low risk of bias, showing an effect on pain, are, furthermore, presented in the following section. According to the described criteria, the evidence for each intervention will following be summarised at the end of each section. 

### 3.3. Effect of Physiotherapy Interventions

#### 3.3.1. Exercise

*Effect of Exercise on Pain in Patients with Chronic Nonspecific Neck Pain.* As shown in Appendix A, Table 1, 18 trials examined the effect of various types of exercise in patients with chronic neck pain; nine of these were at unclear or high risk of bias [31–33, 40–44, 46], and nine were at low risk of bias [34–39, 45, 47, 48]. Seven of the trials at low risk examined the effect of different types of exercise, including proprioception exercises (eye-head coordination), craniocervical flexion exercises (C-CF), neck stabilisation exercises, stretching, strengthening, and behavioural graded activity programme, but did not find statistically significant difference on pain between groups following intervention [34, 36–39, 45, 48]. Two of the trials at low risk of bias succeeded in finding an effect on pain from the intervention, and for this reason, their results were considered evidence of use of exercise.

(1) Gustavsson et al. [35] examined a multicomponent pain and stress self-management group intervention (PASS) versus a control group receiving individually administered physiotherapy (IAPT). There was a statistically significant effect on ability to control pain (*P* < 0.001) and on neck-related disability (NDI) (*P* < 0.001) in favour of PASS at the 20-week followup.
(2) Ylinen et al. [47] examined three interventions: intensive isometric strength training versus lighter endurance training versus a control group. The two training groups had an additional 12-day institutional rehabilitation programme. At the 12-month followup, both neck pain and disability had decreased in both training groups compared with the control group (*P* < 0.01).

No trials with low risk of bias supported single use of proprioception exercises (eye-head co-ordination), cranio-cervical flexion exercises (C-CF), or neck stabilisation exercises for pain. No trials with low risk of bias support the use of stretching.

*Effect of Exercise on Pain in Patients with Chronic Whiplash-Associated Disorder*. As shown in Appendix A, Table 2, seven trials examined the effect of various types of exercise in patients with chronic WAD; three of these were at low risk of bias [49, 51, 55], and four were at unclear or high risk of bias [50, 52–54]. One of the trials at low risk of bias examined the effect of adding biofeedback training to a rehabilitation programme, but found no difference in effect between groups [49]. Two trials at low risk of bias succeeded in finding an effect on pain from the intervention, and for this reason, their results were considered evidence of use of exercise.

(1) Jull et al. [51] examined a multimodal physiotherapy programme (including exercises, education, and ergonomics) versus a self-management programme. The multimodal physiotherapy programme group attained a statistically significant greater reduction in reported neck pain and disability (NDI) posttreatment (*P* = 0.04).
(2) Stewart et al. [55] examined exercise (e.g., endurance, strength, aerobic, coordination, and cognitive behavioural therapy) versus advice alone. Exercise and advice were more effective than advice alone at 6 weeks on pain intensity scale (*P* = 0.005) and on a bothersomeness scale at 6 weeks (*P* = 0.003) and at 12 months (*P* = 0.003).

No trials at low risk of bias support the use of EMG biofeedback.

#### 3.3.2. Manual Therapy

*Effect of Manual Therapy on Pain in Patients with Chronic Nonspecific Neck Pain*. As shown in Appendix A, Table 3, six trials examined the effect of various types of manual therapy in patients with chronic non-specific neck pain [56–61]. One of the trials was at unclear risk of bias, and for that reason not considered evidence [61]. Three trials at low risk of bias examining the effect of spinal manipulations found no difference between groups [56, 58, 60]. Two trials succeeded in finding an effect on pain from the intervention. Both trials had a low risk of bias, and for this reason, their results were considered evidence of use of manual therapy.

(1) Lau et al. [57] examined thoracic manipulation versus a control group. They found statistically significant differences in favour of thoracic manipulation posttreatment on pain intensity (*P* = 0,043) and pain and disability (NPQ) (*P* = 0,018). Improvements were maintained at 3, and 6-month followup.
(2) Sherman et al. [59] examined massage versus a self-care book. They found statistically significant effect on massage following four weeks of treatment on neck pain and disability (NDI) (*P* = 0.047), but not at long-term followup at 10 and 26 weeks.

No trials at low risk of bias support the use of traction. 

#### 3.3.3. Electrotherapy

*Effect of Electrotherapy on Pain in Patients with Chronic Nonspecific Neck Pain*. As shown in Appendix A, Table 4, 11 trials examined the effect of various types of electrotherapy in patients with chronic non-specific neck pain; two of these were at low risk of bias [63, 64], and nine were at unclear or high risk of bias [62, 65–72]. The two trials at low risk of bias both succeeded in demonstrating an effect on pain from this type of intervention and for this reason; their results were considered evidence of use of electrotherapy.

(1) Chiu et al. [63] examined three interventions: TENS versus exercise versus a control group. There were no statistically significant differences between the three groups on pain (VNPS) after 6-week and at 6-month followup, but the TENS group and the exercise group had a significantly better improvement in neck pain and disability (NPQ) than the control group (*P* = 0.034 and *P* = 0.02, resp.) after 6-week, and at 6-month followup.
(2) Chow et al. [64] examined laser versus sham laser treatment. The improvement in VAS was statistically significantly greater in the laser treatment group than in the sham laser treatment group (−2.7 compared with +0.3, *P* < 0.001) at 12-week followup.

No trials at low risk of bias support the use of ultrasound therapy. No trials at low risk of bias support the use of rMS. 

## 4. Discussion

### 4.1. General Interpretation

In this review, we assessed the effect of various interventions for the treatment of chronic neck pain and evaluated the methodological quality of the trials. Our findings emphasise the importance of taking the risk of bias into consideration when evaluating the evidence of an intervention.

Trials varied substantially regarding their internal validity, although the methodological quality of the RCTs in general appeared to be somewhat low with an unclear or high risk of bias. We identified various methodological flaws that may have implications for the internal validity of the trials and consequently may result in biased outcomes. Key domains in this context were randomisation, blinding, and incomplete outcome data. 

Our evaluation also exposes a widespread use of within-group analyses, claiming statistically nonsignificant results to be beneficial. Results were frequently analysed and reported as if they were uncontrolled within-group studies, which consequently led to misinterpretation of results. To some extent this may be due to the absence of a control group in many trials, and the use of an active treatment as a comparative group makes the “proof” of a truly statistically significant effect more difficult to find. We believe that attention should be paid to inadequate interpretation of a trial result when authors inadequately interpret lack of difference in terms of efficacy [73–76]. 

### 4.2. Effect of Physiotherapy on Chronic Neck Pain

Overall, the evidence of effect of physiotherapy for chronic neck pain is strengthened. Yet, for some of the treatments offered, no definite effect and clinical usefulness can be shown. This does not necessarily implicate that these treatments have no effect, only that the present evidence is not sufficient.

Physiotherapy interventions for chronic neck pain showing the strongest support for an effect on pain are strength and endurance training (supported by two trials by Stewart et al. [55] and by Ylinen et al. [47], treating patients with chronic WAD and patients with chronic non-specific neck pain, resp.). In patients with chronic WAD, multimodal physiotherapy was also shown to have a beneficial effect by one trial by Jull et al. [51]. In patients with chronic non-specific neck pain, the use of cognitive/behavioural components in exercise was supported by one trial by Gustavsson et al. [35]. In regard to manual therapy, massage seems to have an effect on pain in patients with chronic non-specific neck pain, supported by one trial by Sherman et al. [59], and thoracic manipulation seems to have an effect on pain, supported by one trial by Lau et al. [57]. Within the area of electrotherapy, both laser therapy and TNS seem to have an effect on pain in patients with chronic non-specific neck pain. This was supported by one trial by Chow et al. [64] and one trial by Chiu et al. [63]. No trials supported the isolated use of proprioception (eye-head co-ordination), cranio-cervical flexion training, stretching, ultrasound therapy, rMS, and traction. 

When looking deeper into the actual components of the various interventions in the above-mentioned trials, four of them—despite the differences, diversity, and individual features of the interventions—seem to have several characteristics in common: The interventions can be considered to be rehabilitative interventions of multimodal physiotherapy with a focus on exercise, including cognitive-behavioural components. This is based on (1) the trial by Stewart et al. [55] showing effect of mixed exercises, where the intervention besides submaximal training, stretching, and aerobic endurance included coordination programme designed to improve functional activities and principles of cognitive behavioural therapy (i.e., setting goals); (2) the trial by Ylinen et al. [47] showing effect of strength training and endurance training, where training groups had an additional 12-day institutional rehabilitation programme with training lessons, behavioural support, ergonomics, sessions of physical manual therapy—including massage/mobilisations—and advice to continue exercise; (3) the trial by Jull et al. [51] showing effect of a multimodal physiotherapy, including low-load exercise for reeducating muscle control of the neck flexor and extensor muscles and scapular muscles, posture exercises, kinaesthetic exercises, and mobilisation techniques, and in addition education including ergonomics, daily living advice, and home exercise; (4) finally, the trial by Gustavsson et al. [35] who found effect from a multi-component pain and stress self-management group intervention—including relaxation training, body awareness exercises, and lectures and group discussions—regarding coping with pain in terms of patients' self-reported pain control, self-efficacy, and disability. Our main results are consistent with findings of previous reviews of interventions for neck pain. The Cochrane Review by Kay et al. [19] on the effect of exercises for mechanical neck disorders concluded that the summarised evidence indicates that there is a role for exercises in the treatment of acute and chronic mechanical neck pain plus headache, but that the relative benefit of each type of exercise needs extensive research. Our review on chronic neck pain agrees with the present conclusion regarding exercise, yet our findings tend to favour strength and endurance training, as well as multimodal physiotherapy in addition to pain and stress self-management. The superior effect of strength training and endurance training may be due to the physical impairments found in the chronic condition [77–80]. 

Our review adds new knowledge regarding the evidence for use of massage. Our findings are in discrepancy to a Cochrane Review by Haraldsson et al. [13] who concluded that the effectiveness of massage for improving neck pain and function remains. Yet the quoted review was last updated in 2004, and the trial by Sherman et al. [59] supporting massage was published in 2009. Our findings on the evidence of manipulation are in line with another Cochrane Review by Gross et al. [18] on the effect of manipulation and mobilisation for neck pain, who found low quality evidence that cervical and thoracic manipulations may provide pain reduction. We too found evidence that thoracic manipulations may have an effect on pain [57]. Regarding low-level laser therapy, our findings are consistent with the findings of a review by Chow et al. [81], who found that low-level laser therapy reduces pain in patients with chronic neck pain. A Cochrane Review from 2007 [17] on the effect of conservative treatment for whiplash concluded that clearly effective treatments are not found for treatment of acute, subacute, or chronic symptoms. The findings of our newer review do support multimodal physiotherapy and mixed exercise programmes for chronic WAD. The explanation for the difference may be that the Cochrane Review by Verhagen in 2007 was not updated after January 2007, and our findings are based on more recent research, namely, two trials published later in 2007 [51, 55]. A more recent review by Teasell et al. [82] found evidence to suggest that exercise programmes are the most effective noninvasive treatments for patients with chronic WAD. Our findings give support to the use of cognitive-behavioural element, and to pain and stress self-management. This is in discrepancy to another Cochrane Review by Gross et al. [14] on patient education for neck pain, concluding that there is no strong evidence for the effectiveness of educational interventions in various neck disorders. This difference may be due to the use of only single-modal trials in their review rather than multimodal trials as used in the current review. 

### 4.3. Strengths and Weaknesses of Review Procedures

To our knowledge, this is the first systematic review on interventions for chronic neck pain addressing the majority of commonly used physiotherapeutic modalities in one study, in order to get an overview of the subject area.

The search strategy and selection criteria we used were quite strict and easy to apply and according to normal procedures for conducting systematic reviews [23]. Yet the following limitations of the literature search may have introduced a bias: some relevant trials may have been missed if they used other keywords, although this is not very likely. We had limitations in language, and this may have led to missing studies from countries in Eastern Europe with a tradition of physiotherapy research, like Poland. We decided to limit our search from 1990 to January 2012. This was due to physiotherapists prior to this time not being trained in scientific methods and also that RCTs were rare. Studies earlier than 1990 would in general not be following a strict protocol like the ones used for RCTs, but at best be longitudinal cohort studies.

The quality assessment was presented in a reproducible manner. However, the results may be affected by our emphases during filtering methods for synthesis evidence. We might have chosen to exclude all trials with insufficient reporting on allocation sequence and allocation concealment. However, we chose not to, since this would have left us with very few trials to assess. We assessed risk of bias, requiring a convincing mechanism to be described in order for a trial to be classified as “adequate.” Our approach to this problem was to assume that the quality was inadequate unless information on the contrary was provided, and in doing so, we might have misclassified well-conducted but badly reported trials. 

The present review succeeded in a subgroup assessment of physiotherapy treatment for chronic non-specific neck pain and for chronic WAD. Yet the first group was very wide due to the mixed conditions in the group of participants. The various interventions were considered to be complex, multifaceted, and with various cointerventions, and by classifying them into intervention groups according to—what we believed to be—the trial's agenda, we may have misclassified some. On the other hand, the often used combined therapies also highlight a fundamental problem when assessing effect of specific and single physiotherapy modalities. Another issue is the quality of the intervention since the interventions were administered in different ways and in different settings. It is reasonable to expect that the way in which they were administered including the dose-response relationship could have influenced the outcome. It would have been interesting and very relevant to examine this. Herbert and Bo [83] emphasise that researchers carrying out systematic reviews should routinely examine the quality of interventions.

### 4.4. Future Directions

We need to know which patients will benefit from which intervention, built on well-conducted and well-reported trials, considering subgroups of patients with chronic neck pain, in order to support recommended evidence-based decisions and to set priorities for future research. We also request future trial investigators to consider to what extent cointerventions are valuable, in addition to possible confounders. Another issue to consider is the extent to which the control groups ought to be given care and attention to the same extent as the intervention groups.

## Figures and Tables

**Table 1:** Exercise therapy—patients with chronic nonspecific neck pain.

Author	Participants	Interventions	Main outcome measures	Study results on effect∗ of intervention on pain
Cunha et al. [31]	Women, aged 35–60, with diagnosed primary mechanical myogenous or arthrogenous, neck pain lasting > 12 wks (*N* = 33)	(1) GPR group (*n* = 15), manual therapy for stretching fasciae for 30 min, muscle stretching in the form of global posture reeducation (GPR) for 30 min (2) Conventional stretching group (*n* = 16), manual therapy for stretching fasciae for 30 min, muscle stretching through conventional stretching exercises for 30 min All: two weekly physiotherapy sessions during a 6 wk period	VAS, ROM, SF-36	There were no statistically significant differences in effect between groups after treatment and at 6 wk followup

Dellve et al. [32]	Women, aged 35–60, with work disability (at least 50%) and pain in the neck (diagnosed cervicobrachial pain syndrome) for at least 1 year (*N* = 60)	(1) Myofeedback training (*n* = 20), min 8 hours/wk, registered the muscle activity (EMG) of upper trapezius muscles and gave alarm if the preset level of muscular rest was not reached. Personal visit once/wk from a physiotherapist browsing EMG profiles with reference to diary entries (2) Intensive muscular strength training (*n* = 20), a structured 5–10 min program to be performed twice a day for 6 days/wk. A physiotherapist coached by two personal visits and additional phone calls twice/wk. (3) Control group (*n* = 20) All: kept a diary 6 days/wk recording activities, discomfort, pain, and sleeping disturbances. All interventions lasted 1 mth	Work ability index (WAI) Single item on work ability, working degree, changed work ability Pain, NRS Copenhagen Psychosocial Questionnaire Cutlery wiping performance test, dexterity, max. grip strength	There were no statistically significant differences in effect between groups after 1 mth and at followup after 3 mths

Falla et al. [33]	Patients with chronic nonsevere neck pain (>3 mths), score < 16 (out of possible 50) in NDI (*N* = 58)	(1) Endurance-strength training of the cervical spine flexor muscles (*n* = 29) (2) Referent exercise intervention, low-load craniocervical exercise (*n* = 29) All: instruction and supervision once a wk for 6 wk, supplied with an exercise diary	EMG measures of maximum voluntary contraction force of sternocleidomastoid and anterior scalene muscle, NRS∗∗, NDI∗∗	There were no statistically significant differences between groups for change in pain (NRS) or disability (NDI) measured in the week immediately after intervention (week 7)

Griffiths et al. [34]	Chronic neck pain (diagnosed spondylosis, whiplash, nonspecific neck pain, and discogenic pain), age 18 and over (*N* = 74)	(1) Specific neck stabilisation exercises (*n* = 37) in addition to the same programme as group 2 (2) General neck exercise programme (*n* = 37), posture correction technique, and active range of movement exercise All: max. four 30 min treatment sessions within the first 6 wks, advice to perform exercises 5–10 times daily, written sheets, after 6 wks the therapist could discharge the patient or continue	NPDS, NPQ, VAS∗∗	There were no significant between-group differences in the NPDS at either 6 wks or 6 mths

Gustavsson et al. [35]	Patients with musculoskeletal tension-type neck pain of persistent duration (>3 mths), age 18–65 (N = 156)	(1) Multicomponent pain and stress self-management group intervention (PASS) (n = 77), relaxation training, body awareness exercises, lectures and group discussions, seven 1.5 h sessions over a 7 wk period, and a booster session after 20 wks (2) Control group receiving individually administered physiotherapy (IAPT) (n = 79)	Questionnaire comprising the self-efficacy scale, NDI, coping strategies questionnaire, hospital and depression scale, fear-avoidance beliefs questionnaire, and questions regarding neck pain, analgesics, and utilisation of health care	There was a statistically significant effect on ability to control pain (P < 0.001), and on neck related disability (NDI) (*P* < 0.001) in favour of PASS at the 20 wks followup

Häkkinen et al. [36]	Nonspecific neck pain of more than 6 mths, age 25–53, pain > 29 mm on VAS (N = 101)	(1) Strength training and stretching (*n* = 49). Sessions once a wk for 6 wks and thereafter one session every second mth for 12 mths (2) Stretching group (*n* = 52) in a single group session instructions All: encouraged to perform home training regimen three times a wk and to keep weekly exercise diary	VAS, neck and shoulder disability index, NDI, ROM, isometric strength	There were no statistically significant differences in effect between groups after two and 12 months measured with VAS and NDI

Jordan et al. [37]	Patients with chronic neck pain (>3 mths), nonradicular extremity pain was permitted, age 20–60 (*N* = 119)	(1) Intensive training of the neck and shoulder musculature (*n* = 40) (2) Individual physiotherapy treatment (*n* = 39) (3) High-velocity, low-amplitude spinal manipulation performed by a chiropractor (*n* = 40) All: above training/treatment sessions twice a wk for 6 wks, besides a single neck school group session	Self-reported disability and pain on 11-point box scales, medication use, patients perceived effect, physicians global assessment	There were no statistically significant differences in effect between groups at 4 and 12 mths followup

Jull et al. [38]	Females with chronic neck pain of idiopathic or traumatic origin and abnormal measures of joint position sense (*N* = 64)	(1) Proprioceptive exercise intervention (*n* = 28) (2) Craniocervical spine flexion exercise intervention (*n* = 30) All: personal instruction and supervision once a wk for 6 wks	Joint position error, NDI, NRS	There were no statistically significant differences in effect between groups measured in the week immediately after intervention (week 7)

Jull et al. [39]	Females with chronic, nonsevere neck pain (>3 mths), score < 15/50 on NDI (*N* = 46)	(1) Craniocervical spine flexion training (*n* = 23), low load (2) Strength training (*n* = 23) All: personal instruction and supervision once a wk for 6 wks	(NDI, NRS)∗∗, EMG amplitude of deep cervical spine flexor muscles, sternocleidomastoid and anterior scalene muscle and ROM	There were no statistically significant differences in effect between groups measured in the week immediately after intervention (week 7)

O'Leary et al. [40]	Females with chronic neck pain (>3 mths), having in the higher end of mild to moderate pain and disability, score > 4/50 on NDI (*N* = 48)	(1) Cranio-cervical spine flexion coordination exercise (CCF) (*n* = 24) (2) Cervical spine flexion endurance exercise (CF) (*n* = 24) All: one experimental session	VAS	There were no statistically significant differences between groups on VAS

Randløv et al. [41]	Females with chronic neck/shoulder pain (>6 mths), age 18–65 (*N* = 77)	(1) Light training (*n* = 41) (2) Intensive training (*n* = 36) All: three times per wk, in total 36 sessions	Pain measures with two 11-point box scales, activities of daily living, strength, endurance	There were no statistically significant differences in effect between groups after six and twelve mths followup

Revel et al. [42]	Patients with chronic neck pain (>3 mths), age > 15 (*N* = 60)	(1) Rehabilitation group (*n* = 30), receiving common symptomatic treatment, besides eye-head exercises improving neck proprioception in individual exercise sessions twice a wk for 8 wks (2) Control group (*n* = 30), receiving only symptomatic treatment without rehabilitation	Head repositioning accuracy, VAS, medication intake, ROM	Significant difference between groups for the rehabilitation group on VAS pain (−21.8 ± 25.2) (P = 0.04) at 10 wk followup

Taimela et al. [43]	Patients with chronic, nonspecific neck pain (>3 mths), half had local pain and half referred pain below the elbow, age 30–60 (*N* = 76)	(1) Active treatment (*n* = 25), proprioceptive exercises, relaxation and behavioural support, 24 sessions (2) Home regimen (*n* = 25), neck lecture and two sessions of practical training for home exercises and instructions for maintaining a diary (3) Control group (*n* = 26), a lecture regarding care of the neck with a recommendation to exercise	VAS, ROM, PPT	The VAS scores after the intervention at 3 mths were significantly lower in the active treatment (22 mm) and home regimen (23 mm) groups than in the control group (39 mm) (P = 0.018) after 3 mths. No statistically significant differences between the groups were noted at 12 mths

Viljanen et al. [44]	Female office workers with chronic non-specific neck pain (>12 wks), age 30–60 (*N* = 393)	(1) Dynamic muscle training (*n* = 135) (2) Relaxation training (*n* = 128) (3) Control group, ordinary activity (*n* = 130) Groups 1 and 2 were instructed and trained 3 times a wk for 12 wks followed by one wk of reinforcement 6 mths after randomisation	Pain rated on a scale 0 (no pain)–10 (unbearable pain), pain questionnaire	There were no statistically significant differences in effect between groups at 3, 6, and 12 mths followup

Vonk et al. [45]	Patients with chronic non-specific neck pain (>3 mths), age 18–70 (N = 139)	(1) Behaviour graded activity programme (*n* = 68), biopsychosocial model guided by the patient's functional abilities (2) Conventional exercise (*n* = 71), reflected usual care, exercises, massage and mobilisation and traction All: treatment period 9 wks	Global perceived effect, NDI, NRS	There were no statistically significant differences in effect between groups at 4, 9, 26, and 52 wks

Waling et al. [46]	Women with chronic work-related trapezius myalgia (>1 ye), not on sick leave more than 1 mth during last year, age < 45 ye (*N* = 103)	(1) Strength training group (*n* = 29), loaded to allow 12 rep. maximum (RM) (2) Endurance training group (*n* = 28), arm-cycling intensity light (11)—somewhat hard (13) on RPE alternating with exercises loaded to 30–35 RM (3) Coordination training (*n* = 25). Body-awareness therapy and training. (4) Control group: nontraining. Group stress and bodily reactions due to stress were studied. Two-hour sessions once a wk for 10 wks Groups 1–3: one-hour sessions, three times a wk for 10 wks	VAS, three scales: pain-in-general, pain-at-worst, pain-at-present. Pain threshold	Significant effect of strength training and endurance training VAS pain-at-worst after 10 wks (P < 0.05). But no difference on VAS pain-at-present or at VAS pain-at-general

Ylinen et al. [47]	Female office worker, age 25–53, with constant or frequently occurring neck pain of more than 6 mths. Motivated to continue working and rehabilitation (N = 180)	(1) Endurance group (n = 60), endurance training, dynamic neck exercises (2) Strength group (n = 60), strength training, high-intensity isometric neck strengthening and stabilisation exercises Groups 1 and 2: 12-day institutional rehabilitation programme with training lessons, behavioural support, 4 sessions of physical manual therapy, advice to continue exercise 3 times a wk at home (3) Control group (n = 60): 3-day institutional rehabilitation programme with recreational activities All: advice to perform aerobic exercise 3 times a wk for half an hour at home	VAS, neck and shoulder pain and disability index, vernon neck disability index	At the 12 mth followup, both neck pain and disability had decreased in both training groups compared with the control group (P < 0.01). Decrease Pain VAS in the endurance group: −35 ((−42)–(−28)); in the strength group: −40 ((−48)–(−32))

Ylinen et al. [48]	Female, age 25–53, with constant or frequently occurring neck pain of more than 6 mths duration, pain > 44 mm on VAS (N = 125)	Crossover trial, after 4 wks (1) Manual therapy group (*n* = 62), low-velocity osteopathic-type mobilisation of cervical joints, traditional massage, passive stretching, two treatments a wk for 4 wks (2) Stretching exercises group (n = 63) consisted of instruction to perform neck stretching exercises at home for 4 wks	VAS, neck and shoulder pain and disability index, NDI,	There were no statistically significant differences in effect between groups at the one- and three-year followup

^*^In order to show an effect of an intervention and hereby support the intervention, it requires showing statistical significant difference between groups.

∗∗Secondary outcome measure.

VAS: visual analogue scale; NRS: numerical rating scale; VNPS: verbal numeric pain scale; NPQ: Northwick Park neck pain questionnaire; NDI: neck disability index; NPDI: neck pain and disability index; NPDS: neck pain and disability scale; NPDVAS: neck pain and disability visual analogue scale; PSFS: patient specific functional scale; NPI: Northwick Park neck pain index; SF-36: short-form 36; PPT: pressure pain threshold; ROM: range of movement; RPE: rating of perceived exertion; EMG: electromyographic, HRQoL: health-related quality of life.

**Table 2:** Exercise therapy—patients with chronic whiplash-associated disorder.

Author	Participants	Interventions	Main outcome measures	Study results on effect∗ of intervention on pain
Ehrenborg and Archenholtz [49]	Patients, aged 17–58, with pain after whiplash injury (>3 mths), and referred to the pain unit for outpatient-based, interdisciplinary rehabilitation (*N* = 65)	(1) Biofeedback training (*n* = 36), eight sessions (twice/wk for four wk) while being active in a self-chosen handicraft. (2) Being active in a self-chosen handicraft on the same terms as group 1 but without biofeedback (*n* = 29). All: 4–6 wk rehabilitation programme consisting of a combination of education, ergonomic interventions, physical training, relaxation techniques, body awareness training, and interventions by psychologist and/or social worker if needed	Canadian occupational performance measure, Multidimensional Pain Inventory, Swedish version	There were no statistically significant differences in effect between groups at 6 mths followup

Fitz-Ritson [50]	Patients with chronic pain in cervical spine musculature following motor vehicle accident (WAD), age 19–57, still having symptoms after receiving chiropractic treatments and rehabilitation exercises for > 12 mths (*N* = 30)	(1) Continued chiropractic treatments and standard rehabilitation exercises (*n* = 15) (2) Continued chiropractic treatments and were advised to do “phasic neck exercises” (eye-head co-ordination) (*n* = 15) All: exercises 5 days a wk for 8 wks	NPDI	The authors do not report any data on statistically significant differences between groups after 8 wk

Jull et al. [51]	Patients with chronic whiplash-associated disorder (>3 mths, <2 yrs), classified WADII, age 18–65 (*N* = 71)	(1) Multimodal physiotherapy programme (MPT) (*n* = 36), low-load exercise for reeducating muscle control of the neck flexor and extensor muscles and scapular muscles, posture exercises, kinaesthetic exercises and mobilisation techniques, education including ergonomics, daily living advice, home exercise (2) Self-management programme, education, advice and exercise (SMP) (*n* = 35) All: intervention period 10 wks	NPI, VAS∗∗	The MPT group attained a statistically significant greater reduction in reported neck pain and disability (NPI) (*P* = 0.04), effect size 0.48, measured immediately following treatment

Pato et al. [52]	Patients with whiplash injury grade I or II (Quebec Task Force Classification), with persistent neck pain or headache 6–12 mths after the accident (*N* = 91)	(1) Local anesthetic infiltration of tender points in the neck 2 × a wk, in 8 wks, (*n* = 30) (2) Physiotherapy, 2 × a wk, in 8 wks: massage, relaxation techniques of myogelotic muscles, instructed in a detailed homeprogram of isometric and low-intensity active isotonic training of neck muscles (*n* = 29) (3) Medication: 200 mg flurbiprofen in its slow release preparation once a day. Patients were seen twice a wk by the same study physician during the 8 wks (*n* = 28) All: furthermore, in each treatment group patients were randomly allocated to additional cognitive-behavioral therapy (CBT) or no CBT. CBT twice a wk for 8 wks. Each session lasted 60 minutes	Subjective outcome rating (free of symptoms, improved, unchanged, worse), McGill pain questionnaire, VAS), working capacity	There were no statistically significant differences between the 3 different treatment groups measured at 8 wk and at 6 mths followup. There was a statistically significant effect in the short term in female patients in the groups with additional CBT (*P* = 0.024) after 8 wks of treatment in the subjective outcome, but not at 6 mths followup

Ryan [53]	Patients with chronic WAD, duration of pain not reported (*N* = 103)	(1) Strength training group (*n* = not reported) (2) Endurance training group (*n* = not reported) All: twice a wk for 8–12 wks	VAS, SF-36, strength	There were no statistically significant differences between groups posttreatment

Söderlund and Lindberg [54]	Patients with chronic WAD, (>3 mths after injury), age 18–60 (*N* = 33)	(1) Physiotherapy with cognitive behavioural components, learning and application of basic physical and psychological skills in everyday activities, besides physiotherapy as in group 2 (*n* = 16) (2) Physiotherapy, individualised exercises at home and/or in departments gym, various pain-relieving methods (i.e., TENS, heat) (*n* = 17) All: max. 12 individual sessions with the physiotherapist	PDI, NRS, physical measures of pain, disability, coping and self-efficacy	Results revealed no statistically significant differences between groups in self-ratings of disability or pain intensity post treatment or at 3 mths followup

Stewart et al. [55]	Patients with chronic WAD (>3 mths, <12 mths), classified WAD I–III, having significant pain or disability (*N* = 134)	(1) Advice alone group (*n* = 68), received education, reassurance and encouragement to participate in light activity alone, advice given in one consultation and two follow-up phone contacts (2) Advice and exercise group (*n* = 66), individualised, progressive, submaximal programme designed to improve functional activities, endurance, strength, aerobic, speed, coordination, principles of cognitive behavioral therapy (i.e., setting goals), 12 sessions during 6 wks	Pain intensity and pain bothersomeness rated on a 0–10 box scale, PSFC	Exercise and advice were more effective than advice alone at 6 wks for all primary outcomes but not at 12 months. The effect of exercise on the 0–10 pain intensity scale was −1.1 (95% CI −1.8 to −0.3, *P* = 0.005) at 6 wks and −0.2 (0.6 to −1.0, *P* = 0.59) at 12 mths; on the bothersomeness scale the effect was −1.0 (−1.9 to −0.2, *P* = 0.003) at 6 wks and 0.3 (−0.6 to 1.3, *P* = 0.48) at 12 mths

^*^In order to show an effect of an intervention and hereby support the intervention, it requires showing statistical significant difference between groups.

∗∗Secondary outcome measure.

VAS: visual analogue scale; NRS: numerical rating scale; VNPS: verbal numeric pain scale; NPQ: Northwick Park neck pain questionnaire; NDI: neck disability index; NPDI: neck pain and disability index; NPDS: neck pain and disability scale; NPDVAS: neck pain and disability visual analogue scale; PSFS: patient-specific functional scale; NPI: Northwick Park neck pain index; SF-36: short-form 36; PPT: pressure pain threshold; ROM: range of movement; RPE: rating of perceived exertion; EMG: electromyographic, HRQoL: health-related quality of life.

**Table 3:** Manual therapy—patients with chronic nonspecific neck pain.

Author	Participants	Interventions	Main outcome measures	Study results on effect∗ of intervention between groups
Bronfort et al. [56]	Patients with mechanical neck pain lasting > 12 wks, age 20–65 (*N* = 191)	(1) Spinal manipulation and low-technology rehabilitative neck exercise (*n* = 63) (2) High-technology MedX rehabilitative neck exercise (*n* = 60) (3) Spinal manipulation (*n* = 64) All: attended 20 one-hour visits during the 11 wk study	Pain rating scale (0–10), NDI, SF-36, global improvement of satisfaction with care, medication use	No statistically significant differences between groups in patient rated outcomes at 11 wk and at 12 mth followup

Lau et al. [57]	Patients with a diagnosis of chronic mechanical neck pain (>3 mths), age 18–55 (*N* = 120)	(1) Thoracic manipulations TM, anterior-posterior approach in supine lying (*n* = 60) (2) Control (*n* = 60) All: 8 sessions infrared radiation (2/wk) for 15 min over painful site. Educational pamphlet involving active neck mobilisation, isometric neck muscle stabilisation, stretching, postural correction exercise	NPRS, NPQ, SF-36, cervical ROM, craniovertebral angle	Statistically significant differences in favour of TM post-treatment on pain intensity (*P* = 0.043) and NPQ (*P* = 0.018). Improvements were maintained at 3 and 6 mths followup

Martel et al. [58]	Patients with pain of mechanical origin located in the anatomical region of the neck, with or without radiation to the head, trunk, or limbs > 12 wks; between 18 and 60 yrs (*N* = 98)	All: spinal manipulation 10–15 treatments in 5-6 wk (symptomatic phase) after that 3 different interventions (preventive phase). (1) Spinal manipulation cervical and thoracic until Th4, once per month, 4 times (*n* = 36) (2) Spinal manipulation cervical and thoracic until Th4, once per mth, 4 times AND 20–30 min home exercises 3× per wk: including range of motion exercises, 4 stretching/mobilisation, and 4 strengthening exercises. Three series of each exercise. Ten mths (*n* = 33) (3) Attention control group: no treatment (*n* = 29)	VAS, cervical ROM, NPDI, Bournemouth questionnaire, SF-12 questionnaire, fear-avoidance behaviour questionnaire	No statistically significant differences were found between groups

Sherman et al. [59]	Patients with chronic neck pain (>3 mths), age 20–64 (*N* = 64)	(1) Therapeutic neck massage (*n* = 32), commonly used Swedish and clinical massage techniques, allowed typical self-care recommendations, up to 10 treatments over a 10 wk period (2) Self-care book (*n* = 32), they were mailed a copy of a self-care book with information and recommendation	NDI, NRS	Statistically significant effect on massage after four wks measured by NDI, −2.1 (−4.00–0.03) (*P* = 0.047), but not in long-term followup at 10 and 26 wks

Sillevis et al. [60]	Patients between 18 and 65 from outpatients physiotherapy clinic with non-specific pain in the cervical and cervicothoracic region down to T4, provoked with neck movements, present for at least 3 mths (*N* = 100)	(1) One time thrust manipulation at T3-T4 (*n* = 50) (2) Placebo manipulation at T3-T4 (*n* = 50)	VAS, pupil diameter	No statistically significant differences between groups immediately after the treatment

Yağci et al. [61]	Patients with chronic cervical myofascial pain syndrome (>6 mths), age 21–44 (*N* = 40)	(1) Spray-stretch technique (*n* = 20), ethyl chloride sprayed on muscle with trigger point in muscle stretched position, 6 sessions. (2) Connective tissue massage (*n* = 20), 15 sessions All: followed active exercises to be carried out three times a day	VAS, pain threshold, ROM, strength, endurance	No statistically significant differences between groups were found on pain posttreatment

^*^In order to show an effect of an intervention and hereby support the intervention, it requires showing statistical significant difference between groups.

VAS: visual analogue scale; NRS: numerical rating scale; VNPS: verbal numeric pain scale; NPQ: Northwick Park neck pain questionnaire; NDI: neck disability index; NPDI: neck pain and disability index; NPDS: neck pain and disability scale; NPDVAS: neck pain and disability visual analogue scale; PSFS: patient-specific functional scale; NPI: Northwick Park neck pain index; SF-36: short-form 36; PPT: pressure pain threshold; ROM: range of movement; RPE: rating of perceived exertion; EMG: electromyographic, HRQoL: health-related quality of life.

**Table 4:** Electrotherapy—patients with chronic nonspecific neck pain.

Author	Participants	Interventions	Main outcome measures	Study results on effect∗ of intervention on pain
Altan et al. [62]	Patients with chronic cervical myofascial pain syndrome (>3 mths), having tender points (*N* = 53)	(1) Laser treatment (*n* = 23), applied over four trigger points bilat., frequency 1000 Hz for 2 min over each point. Laser parameters: infrared 27 GaAs diode, 904 nm, frequency range 5–7000 Hz, max power of 27 W, 50 W, or 27 × 4 W was used (2) Placebo, sham laser treatment (*n* = 25) All: treatment once a day for 10 days during a period of 14 days, instructed to perform isometric exercises and stretching at home	VAS, algometric measurements, ROM	There were no significant differences between groups immediately after (wk 2) and at 12 wks followup

Chiu et al. [63]	Patients with chronic intermittent neck pain (>3 mths), age 20–70 (*N* = 218)	(1) TENS group (*n* = 73): infrared radiation, advice on neck care, TENS to the neck region for 30 min. TENS parameters: dual-channel TENS unit (130 Z), continuous 150 *μ*s square pulses at 80 Hz, four surface electrodes, intensity of TENS was adjusted to produce a tingling sensation (2) Exercise group (*n* = 67): infrared radiation, advice on neck care, intensive neck exercise programme. (3) Control group (*n* = 78): infrared radiation, advice on neck care. All: two sessions a wk for six wks	VNPS, NPQ, NPI, strength	There were no statistically significant differences between the three groups on VNPS pain after 6 wk and at 6 mths followup, but the TENS group and the exercise group had a significantly better improvement in NPQ than that of the control group (*P* = 0.034 and *P* = 0.02, resp.) after 6 wks and at 6 mths followup

Chow et al. [64]	Patients with chronic neck pain (>3 mths), age > 18 (*N* = 90)	(1) Laser treatment (*n* = 45), applied to tender points for 30 s per point with up to 50 points being treated. Laser parameters: class 3B, diolase devices, wavelength 830 nm, power of 300 mW in continuous wave mode at a power density of 0.67 W/cm^2^ (2) Sham laser treatment (*n* = 45) All: 14 treatments over 7 wks	VAS	The improvement in raw VAS was statistically significantly greater in the laser-treatment group than in the sham laser treatment group (−2.7 compared with +0.3, *P* < 0.001). at 12 wk followup

Dundar et al. [65]	Patients with chronic cervical myofascial pain, having spot tenderness along taut band, age 20–60 (*N* = 64)	(1) Laser treatment (*n* = 32), applied over three trigger points bilat., frequency 1,000 Hz for 2 min over each point, power output 58 mW/cm^2^ by 1,000 Hz. Dose per point 7 J, total per treatment 42 J. Laser parameters: infrared Ga-As-Al diode, wavelength 830 nm, max power output of 450 mW (2) Placebo, sham laser (*n* = 32) All: once a day for 15 days during 3 wks, instructed in daily isometric exercise and stretching exercise	VAS, ROM, NDI	There were no statistically significant differences between groups after 4 wks

Esenyel et al. [66]	Patients with chronic myofascial trigger points (duration 6 months to 7 yrs) in one side of the upper trapezius muscles (*N* = 102)	(1) Ultrasound therapy (*n* = 36), dose 1.5 W/cm^2^, 6 min, 10 sessions (2) Trigger point injections (1% lidocaine) (*n* = 36) (3) Control (*n* = 30) All: neck-stretching exercises	VAS, PPT, ROM	Statistically significant and equal reduction in VAS pain from ultrasound and injection groups compared with controls (*P* < 0.001) after treatment and at 3 mth followup. There were no statistically significant differences in outcome measures between groups 1 and 2

Gam et al. [67]	Patients with chronic trigger points in the neck and with an intensity disturbing normal daily activity, age 18–60 (*N* = 67)	(1) Ultrasound, massage, exercise (*n* = 18), dose 100 Hz, pulse = 2 : 8, 3 W/cm^2^, 3 min (2) Sham ultrasound, massage, exercise (*n* = 22) (3) Control group (*n* = 18) Groups 1 and 2 were treated 2 sessions per wk in 4 wks	VAS, measure of trigger points	There were no significant differences between groups post treatment and at 6 mth followup

Gur et al. [68]	Patients with chronic myofascial pain syndrome in the neck (>1 yr), affecting quality of life, with 1–10 tender points in shoulder girdle (*N* = 60)	(1) Laser treatment (*n* = 30), 2 J/cm^2^ at each trigger point (max. 20 J/cm^2^). Laser parameters: Ga-As laser, 20 W max output per pulse, 904 nm, 200 nanoseconds max duration pulse, 2,8 kHz pulse frequency, 11.2 mW average power, 1 cm^2^ surface (2) Placebo, sham laser treatment (*n* = 30) All: treatment 3 min at each triggerpoint, 5 times a wk for 2 wks, instructed in correct posture, ergonomics and to avoid activity exacerbated pain	NPDS, VAS	Statistically significant difference on pain in favour of laser treatment at 2nd wk and 3rd wk on pain VAS (2nd wk: VAS pain at rest 3.11 ± 2.29, *P* = 0.01; VAS pain at movement 2.67 ± 2.58, *P* = 0.01) and NPDS, and at 12 wk followup maintained at NPDS (41.14 ± 28.34) (*P* = 0.01)

Özdemir et al. [69]	Patients with chronic neck pain related to osteoarthritis (*N* = 60)	(1) Low-level laser therapy (*n* = 30), applied to 12 points, 0.90 J for each 1 cm^2^, each point for 15 s. Laser parameters: endolaser 476, Ga-As_Al, power output of 50 mW, wavelength 830 nm, diameter beam 1 mm., 0.90 J for each 1 cm^2^ (2) Placebo, sham laser (*n* = 30) All: treatment in 10 consecutive days	VAS, physician assessment of pressure pain, angle of lordosis, ROM, NPDS	The authors did not report any data on statistically significant differences on pain between groups after treatment

Seidel and Uhlemann [70]	Patients with chronic cervical pain syndrome (>6 mths) (*N* = 51)	(1) Placebo, sham laser treatment (*n* = 13) (2) Laser treatment (*n* = 12), output 7 mW, stimulation to meridian points, 1 min per point, totally 15 points. Laser parameters: cw-IR-GaAIAs-Laser, 830 nm, Lasotronic, energy density 0 J/cm^2^; 21 J/cm^2^; 90 J/cm^2^, irradiation area 0.02 cm^2^, laser skin difference 8 mm (3) Laser treatment (*n* = 13), output 30 mW, stimulation to meridian points, 1 min per point, totally 15 points. Laser parameters: cw-IR-GaAIAs-Laser, 830 nm, Lasotronic, energy density 0 J/cm^2^; 21 J/cm^2^; 90 J/cm^2^, irradiation area 0.02 cm^2^, laser skin difference 8 mm (4) Needle acupuncture (*n* = 13) All: 8 treatments in 4 wk	VAS, PPT, ROM	The authors did not report any data on statistically significant differences on pain between groups after 4 wk

Smania et al. [71]	Patients with chronic myofascial pain syndrome of the superior trapezius muscle (and in no other muscle), age 18–80 (*N* = 53)	(1) Repetitive magnetic stimulation (rMS) (*n* = 17), stimulation to trigger points with figure-eight-shaped coil until coil temperature reached 40 degrees and then replaced by circular coil, pulsed magnetic stimuli (4000) each 20 min session in 5-second trains at 20 Hz separated by 25-second pause. (2) Transcutaneous electrical stimulation (TENS) (*n* = 18), 100 Hz, pulse width 250 *μ*s, asymmetrical rectangular biphasic wave form, intensity set to patients comfort until significant local sensation (3) Placebo (*n* = 18), sham-ultrasound therapy All: treatment sessions, 2 times a wk for 2 wks	NPDVAS, VAS, PPT, ROM	The rMS group and the TENS group showed a statistically significant improvement in the NPDVAS compared to the placebo group: differences to placebo group in NPDVAS, rMS group: pre-post *P* < 0.01; pre-1 mth *P* < 0.001; pre-3 mths *P* = 0.038. Differences to placebo group in NPDVAS, TENS group: pre-post *P* < 0.01; no difference in pre-1 mths and pre-3 mths test. Difference in effect on NPDVAS between rMS and TNS in favour of rMS only in pre-1 mths test, *P* < 0.001, and in pre-3 mths test, *P* < 0.001

Thorsen et al. [72]	Female laboratory workers with chronic pain (>1 yr) from neck and shoulder girdle, pain affecting the quality of work or daily living, 1–10 tender points, age 18–65 yrs (*N* = 49)	Crossover study, 6 sessions over 2 wks followed by one wk pause before 6 new treatment sessions over 2 wks in other group. (1) Laser treatment (*n* = 25) 0.9 J per treated point max 9 J per treatment. Laser parameters: endolaser 465 class no. B, 830 nm ± 0.5 nm, 30 mW, Ga_AI_As diode, beam divergence 4 degrees, probehead 2.5 mm^2^ (2) Placebo, sham laser treatment (*n* = 22) All: 6 sessions over a 2 wk period	VAS	There were no statistically significant differences between groups post treatment

^*^In order to show an effect of an intervention and hereby support the intervention, it requires showing statistical significant difference between groups.

VAS: visual analogue scale; NRS: numerical rating scale; VNPS: verbal numeric pain scale; NPQ: Northwick Park neck pain questionnaire; NDI: neck disability index; NPDI: neck pain and disability index; NPDS: neck pain and disability scale; NPDVAS: neck pain and disability visual analogue scale; PSFS: patient-specific functional scale; NPI: Northwick Park neck pain index; SF-36: short-form 36; PPT: pressure pain threshold; ROM: range of movement; RPE: rating of perceived exertion; EMG: electromyographic, HRQoL: health-related quality of life.

**Table:** (a)

Author	Agenda	Sequence generation	Allocation concealment	Blinding of participants, personnel, and outcome assessors	Incomplete outcome data	Selective outcome reporting	Other sources of bias	Result of summary assessment of risk of bias
Cunha et al. [31]	Global posture reeducation + stretching	Adequate	Unclear	Inadequate	Inadequate	Unclear	Unclear	High
Dellve et al. [32]	Myofeedback training + intensive strength training	Unclear	Unclear	Unclear	Inadequate	Unclear	Unclear	Unclear
Ehrenborg and Archenholtz [49]	Biofeedback training + interdisciplinary rehabilitation	Adequate	Adequate	Inadequate	Adequate	Unclear	Unclear	Low
Falla et al. [33]	Endurance-strength exercise	Adequate	Unclear	Inadequate	Adequate	Unclear	Adequate	Unclear
Fitz-Ritson [50]	Proprioception, eye-head-neck coordination	Inadequate	Inadequate	Inadequate	Inadequate	Unclear	Unclear	High
Griffiths et al. [34]	Specific neck stabilisation exercises + general exercises	Adequate	Adequate	Inadequate	Adequate	Unclear	Adequate	Low
Gustavsson et al. [35]	Multicomponent pain and stress self-management group intervention + individual physiotherapy	Adequate	Adequate	Inadequate	Adequate	Unclear	Adequate	Low
Häkkinen et al. [36]	Strength training + stretching	Adequate	Adequate	Inadequate	Adequate	Unclear	Adequate	Low
Jordan et al. [37]	Intensive training + physiotherapy + chiropractic manipulation	Adequate	Adequate	Inadequate	Unclear	Unclear	Adequate	Low
Jull et al. [38]	Proprioception, eye-head coordination + cranio-cervical flexion	Adequate	Adequate	Inadequate	Inadequate	Unclear	Adequate	Low
Jull et al. [51]	Multimodal physiotherapy programme	Adequate	Adequate	Inadequate	Adequate	Unclear	Adequate	Low
Jull et al. [39]	Cranio-cervical flexion exercise + strength exercises	Adequate	Unclear	Inadequate	Adequate	Unclear	Adequate	Low
O'Leary et al. [40]	Cranio-cervical flexion + cervical flexion endurance training	Unclear	Unclear	Inadequate	Unclear	Unclear	Adequate	Unclear
Pato et al. [52]	Cognitive behavioural therapy + multimodal physiotherapy + infiltration + medication	Unclear	Unclear	Inadequate	Inadequate	Unclear	Unclear	Unclear
Randløv et al. [41]	Intensive training + light training	Adequate	Unclear	Inadequate	Unclear	Unclear	Adequate	Unclear
Revel et al. [42]	Proprioception, eye-head-neck coordination	Unclear	Unclear	Inadequate	Inadequate	Unclear	Adequate	Unclear
Ryan [53]	Strength training + endurance training	Unclear	Unclear	Inadequate	Inadequate	Unclear	Unclear	High
Söderlund and Lindberg [54]	Cognitive behavioural programme	Unclear	Unclear	Inadequate	Adequate	Unclear	Adequate	Unclear
Stewart et al. [55]	Exercise	Adequate	Adequate	Inadequate	Adequate	Adequate	Adequate	Low
Taimela et al. [43]	Multimodal proprioceptive training + home exercises	Unclear	Unclear	Inadequate	Adequate	Unclear	Adequate	Unclear
Viljanen et al. [44]	Dynamic muscle training + relaxation training	Adequate	Unclear	Inadequate	Adequate	Unclear	Adequate	Unclear
Vonk et al. [45]	Behavioural graded activity + exercise	Adequate	Adequate	Inadequate	Adequate	Unclear	Adequate	Low
Waling et al. [46]	Strength + endurance + coordination training	Unclear	Unclear	Inadequate	Inadequate	Unclear	Adequate	High
Ylinen et al. [47]	Intensive strength training + lighter endurance training	Adequate	Adequate	Inadequate	Adequate	Unclear	Adequate	Low
Ylinen et al. [48]	Stretching exercises + manual therapy	Adequate	Adequate	Inadequate	Adequate	Unclear	Adequate	Low

**Table:** (b)

Author	Agenda	Sequence generation	Allocation concealment	Blinding of participants, personnel, and outcome assessors	Incomplete outcome data	Selective outcome reporting	Other sources of bias	Result of summary assessment of risk of bias
Bronfort et al. [56]	Manipulation + exercise	Adequate	Adequate	Inadequate	Adequate	Unclear	Adequate	Low
Lau et al. [57]	Thoracic manipulation	Adequate	Adequate	Inadequate	Adequate	Unclear	Unclear	Low
Martel et al. [58]	Spinal manipulation + home exercise	Adequate	Adequate	Inadequate	Adequate	Adequate	Adequate	Low
Sherman et al. [59]	Massage	Adequate	Adequate	Inadequate	Adequate	Unclear	Adequate	Low
Sillevis et al. [60]	Thoracic manipulation	Adequate	Adequate	Inadequate	Adequate	Unclear	unclear	Low
Yağci et al. [61]	Connective tissue massage + spray-stretch technique	Unclear	Unclear	Inadequate	Unclear	Unclear	Unclear	Unclear

**Table:** (c)

Author	Agenda	Sequence generation	Allocation concealment	Blinding of participants, personnel, and outcome assessors	Incomplete outcome data	Selective outcome reporting	Other sources of bias	Result of summary assessment of risk of bias
Altan et al. [62]	Laser	Unclear	Unclear	Adequate	Inadequate	Unclear	Unclear	Unclear
Chiu et al. [63]	TENS	Adequate	Adequate	Inadequate	Adequate	Unclear	Adequate	Low
Chow et al. [64]	Laser	Adequate	Adequate	Adequate	Adequate	Unclear	Unclear	Low
Dundar et al. [65]	Laser	Adequate	Unclear	Inadequate	Adequate	Unclear	Unclear	Unclear
Esenyel et al. [66]	Ultrasound	Unclear	Unclear	Inadequate	Unclear	Unclear	Unclear	Unclear
Gam et al. [67]	Ultrasound	Adequate	Unclear	Adequate	Inadequate	Unclear	Adequate	Unclear
Gur et al. [68]	Laser	Adequate	Unclear	Inadequate	Unclear	Unclear	Unclear	Unclear
Özdemiret al. [69]	Laser	Unclear	Unclear	Unclear	Unclear	Unclear	Unclear	Unclear
Seidel and Uhlemann [70]	Laser	Adequate	Unclear	Inadequate	Unclear	Unclear	Unclear	Unclear
Smania et al. [71]	rMS + TENS	Adequate	Unclear	Inadequate	Unclear	Adequate	Unclear	Unclear
Thorsen et al. [72]	Laser	Unclear	Unclear	Adequate	Inadequate	Unclear	Adequate	Unclear

## Appendices

### A. Study Characteristics and Study Results

See Tables 1–4.

### B. Risk of Bias

See Table 5.

## References

[1] Becker N., Thomsen A. B., Olsen A. K., Sjøgren P., Bech P., Eriksen J. (1997). Pain epidemiology and health related quality of life in chronic non-malignant pain patients referred to a Danish multidisciplinary pain center. *Pain*.

[2] Borghouts J. A. J., Koes B. W., Vondeling H., Bouter L. M. (1999). Cost-of-illness of neck pain in The Netherlands in 1996. *Pain*.

[3] Brooks P. M. (2006). The burden of musculoskeletal disease—a global perspective. *Clinical Rheumatology*.

[4] Kjoller M., Juel K., Kamper-Jorgensen F. (2009). *Folkesundhedsrapporten Danmark 2007*.

[5] Hogg-Johnson S., van der Velde G., Carroll L. J. (2008). The burden and determinants of neck pain in the general population: results of the Bone and Joint Decade 2000–2010 Task Force on Neck Pain and Its Associated Disorders. *Spine*.

[6] Carroll L. J., Holm L. W., Hogg-Johnson S. (2009). Course and prognostic factors for neck pain in whiplash-associated disorders (WAD). Results of the Bone and Joint Decade 2000–2010 task force on Neck Pain and Its Associated Disorders. *Journal of Manipulative and Physiological Therapeutics*.

[7] Berglund A., Bodin L., Jensen I., Wiklund A., Alfredsson L. (2006). The influence of prognostic factors on neck pain intensity, disability, anxiety and depression over a 2-year period in subjects with acute whiplash injury. *Pain*.

[8] Boersma K., Linton S. J. (2006). Expectancy, fear and pain in the prediction of chronic pain and disability: a prospective analysis. *European Journal of Pain*.

[9] Fejer R., Hartvigsen J. (2008). Neck pain and disability due to neck pain: what is the relation?. *European Spine Journal*.

[10] Harvey N., Cooper C. (2005). Physiotherapy for neck and back pain. *British Medical Journal*.

[11] Soderlund A., Lindberg P. (1999). Long-term functional and psychological problems in whiplash associated disorders. *International Journal of Rehabilitation Research*.

[12] Borghouts J., Janssen H., Koes B., Muris J., Metsemakers J., Bouter L. (1999). The management of chronic neck pain in general practice. A retrospective study. *Scandinavian Journal of Primary Health Care*.

[13] Haraldsson B. G., Gross A. R., Myers C. D. (2006). Massage for mechanical neck disorders. *Cochrane Database of Systematic Reviews*.

[14] Gross A., Forget M., St George K. (2012). Patient education for neck pain. *Cochrane Database of Systematic Reviews*.

[15] Kroeling P., Gross A., Goldsmith C. H. (2009). Electrotherapy for neck pain. *Cochrane Database of Systematic Reviews*.

[16] Graham N., Gross A., Goldsmith C. H. (2008). Mechanical traction for neck pain with or without radiculopathy. *Cochrane Database of Systematic Reviews*.

[17] Verhagen A. P., Scholten-Peeters G. G., van Wijngaarden S., de Bie R. A., Bierma-Zeinstra S. M. (2007). Conservative treatments for whiplash. *Cochrane Database of Systematic Reviews*.

[18] Gross A., Miller J., D'Sylva J. (2010). Manipulation or mobilisation for neck pain: a Cochrane Review. *Manual Therapy*.

[19] Kay T. M., Gross A., Goldsmith C. (2005). Exercises for mechanical neck disorders. *Cochrane Database of Systematic Reviews*.

[20] Guyatt G. H., Oxman A. D., Vist G. E. (2008). GRADE: an emerging consensus on rating quality of evidence and strength of recommendations. *British Medical Journal*.

[21] Kuorinka I., Jonsson B., Kilbom A. (1987). Standardised Nordic questionnaires for the analysis of musculoskeletal symptoms. *Applied Ergonomics*.

[22] Christensen R., Bliddal H. (2010). Is Phytalgic a goldmine for osteoarthritis patients or is there something fishy about this nutraceutical? A summary of findings and risk-of-bias assessment. *Arthritis Research and Therapy*.

[23] Higgins J. P. T., Green S. (2008). *Cochrane handbook for systematic reviews of interventions Version 5.0.1*.

[24] Liberati A., Altman D. G., Tetzlaff J. (2009). The PRISMA statement for reporting systematic reviews and meta-analyses of studies that evaluate health care interventions: explanation and elaboration. *Annals of Internal Medicine*.

[25] Bartels E. M. (2009). How to keep up with medical literature. *Best Practice and Research*.

[26] Resnick D. N. (2005). Subjective outcome assessments for cervical spine pathology: a narrative review. *Journal of Chiropractic Medicine*.

[27] Ware J. E. (2000). SF-36 health survey update. *Spine*.

[28] de Koning C. H. P., van den Heuvel S. P., Staal J. B., Smits-Engelsman B. C. M., Hendriks E. J. M. (2008). Clinimetric evaluation of methods to measure muscle functioning in patients with non-specific neck pain: a systematic review. *BMC Musculoskeletal Disorders*.

[29] de Koning C. H. P., van den Heuvel S. P., Staal J. B., Smits-Engelsman B. C. M., Hendriks E. J. M. (2008). Clinimetric evaluation of active range of motion measures in patients with non-specific neck pain: a systematic review. *European Spine Journal*.

[30] Juul-Kristensen B., Kadefors R., Hansen K., Byström P., Sandsjö L., Sjøgaard G. (2006). Clinical signs and physical function in neck and upper extremities among elderly female computer users: the NEW study. *European Journal of Applied Physiology*.

[31] Cunha A. C. V., Burke T. N., França F. J. R., Marques A. P. (2008). Effect of global posture reeducation and of static stretching on pain, range of motion, and quality of life in women with chronic neck pain: a randomized clinical trial. *Clinics*.

[32] Dellve L., Ahlstrom L., Jonsson A. (2011). Myofeedback training and intensive muscular strength training to decrease pain and improve work ability among female workers on long-term sick leave with neck pain: a randomized controlled trial. *International Archives of Occupational and Environmental Health*.

[33] Falla D., Jull G., Hodges P., Vicenzino B. (2006). An endurance-strength training regime is effective in reducing myoelectric manifestations of cervical flexor muscle fatigue in females with chronic neck pain. *Clinical Neurophysiology*.

[34] Griffiths C., Dziedzic K., Waterfield J., Sim J. (2009). Effectiveness of specific neck stabilization exercises or a general neck exercise program for chronic neck disorders: a randomized controlled trial. *The Journal of Rheumatology*.

[35] Gustavsson C., Denison E., Koch L. V. (2010). Self-management of persistent neck pain: a randomized controlled trial of a multi-component group intervention in primary health care. *European Journal of Pain*.

[36] Häkkinen A., Kautiainen H., Hannonen P., Ylinen J. (2008). Strength training and stretching versus stretching only in the treatment of patients with chronic neck pain: a randomized one-year follow-up study. *Clinical Rehabilitation*.

[37] Jordan A., Bendix T., Nielsen H., Hansen F. R., Høst D., Winkel A. (1998). Intensive training, physiotherapy, or manipulation for patients with chronic neck pain: a prospective, single-blinded, randomized clinical trial. *Spine*.

[38] Jull G., Falla D., Treleaven J., Hodges P., Vicenzino B. (2007). Retraining cervical joint position sense: the effect of two exercise regimes. *Journal of Orthopaedic Research*.

[39] Jull G. A., Falla D., Vicenzino B., Hodges P. W. (2009). The effect of therapeutic exercise on activation of the deep cervical flexor muscles in people with chronic neck pain. *Manual Therapy*.

[40] O'Leary S., Falla D., Hodges P. W., Jull G., Vicenzino B. (2007). Specific therapeutic exercise of the neck induces immediate local hypoalgesia. *Journal of Pain*.

[41] Randløv A., Østergaard M., Manniche C. (1998). Intensive dynamic training for females with chronic neck/shoulder pain. A randomized controlled trial. *Clinical Rehabilitation*.

[42] Revel M., Minguet M., Gergoy P., Vaillant J., Manuel J. L. (1994). Changes in cervicocephalic kinesthesia after a proprioceptive rehabilitation program in patients with neck pain: a randomized controlled study. *Archives of Physical Medicine and Rehabilitation*.

[43] Taimela S., Takala E. P., Asklöf T., Seppälä K., Parviainen S. (2000). Active treatment of chronic neck pain: a prospective randomized intervention. *Spine*.

[44] Viljanen M., Malmivaara A., Uitti J., Rinne M., Palmroos P., Laippala P. (2003). Effectiveness of dynamic muscle training, relaxation training, or ordinary activity for chronic neck pain: randomised controlled trial. *British Medical Journal*.

[45] Vonk F., Verhagen A. P., Twisk J. W., Köke A. J. A., Luiten M. W. C. T., Koes B. W. (2009). Effectiveness of a behaviour graded activity program versus conventional exercise for chronic neck pain patients. *European Journal of Pain*.

[46] Waling K., Sundelin G., Ahlgren C., Järvholm B. (2000). Perceived pain before and after three exercise programs—a controlled clinical trial of women with work-related trapezius myalgia. *Pain*.

[47] Ylinen J., Takala E. P., Nykanen M. (2003). Active neck muscle training in the treatment of chronic neck pain in women: a randomized controlled trial. *The Journal of the American Medical Association*.

[48] Ylinen J., Kautiainen H., Wirén K., Häkkinen A. (2007). Stretching exercises vs manual therapy in treatment of chronic neck pain: a randomized, controlled cross-over trial. *Journal of Rehabilitation Medicine*.

[49] Ehrenborg C., Archenholtz B. (2010). Is surface EMG biofeedback an effective training method for persons with neck and shoulder complaints after whiplash-associated disorders concerning activities of daily living and pain—a randomized controlled trial. *Clinical Rehabilitation*.

[50] Fitz-Ritson D. (1995). Phasic exercises for cervical rehabilitation after “whiplash” trauma. *Journal of Manipulative and Physiological Therapeutics*.

[51] Jull G., Sterling M., Kenardy J., Beller E. (2007). Does the presence of sensory hypersensitivity influence outcomes of physical rehabilitation for chronic whiplash?—a preliminary RCT. *Pain*.

[52] Pato U., di Stefano G., Fravi N. (2010). Comparison of randomized treatments for late whiplash. *Neurology*.

[53] Ryan J. M. (2002). *Reducing Pain and Disability for Whipiash Victims: A Double-Blind Randomised Controlled trial*.

[54] Söderlund A., Lindberg P. (2001). Cognitive behavioural components in physiotherapy management of chronic whiplash associated disorders (WAD)—a randomised group study. *Physiotherapy Theory and Practice*.

[55] Stewart M. J., Maher C. G., Refshauge K. M., Herbert R. D., Bogduk N., Nicholas M. (2007). Randomized controlled trial of exercise for chronic whiplash-associated disorders. *Pain*.

[56] Bronfort G., Evans R., Nelson B., Aker P. D., Goldsmith C. H., Vernon H. (2001). A randomized clinical trial of exercise and spinal manipulation for patients with chronic neck pain. *Spine*.

[57] Lau H. M. C., Wing Chiu T. T., Lam T. H. (2011). The effectiveness of thoracic manipulation on patients with chronic mechanical neck pain—a randomized controlled trial. *Manual Therapy*.

[58] Martel J., Dugas C., Dubois J. D., Descarreaux M. (2011). A randomised controlled trial of preventive spinal manipulation with and without a home exercise program for patients with chronic neck pain. *BMC Musculoskeletal Disorders*.

[59] Sherman K. J., Cherkin D. C., Hawkes R. J., Miglioretti D. L., Deyo R. A. (2009). Randomized trial of therapeutic massage for chronic neck pain. *Clinical Journal of Pain*.

[60] Sillevis R., Cleland J., Hellman M., Beekhuizen K. (2010). Immediate effects of a thoracic spine thrust manipulation on the autonomic nervous system: a randomized clinical trial. *Journal of Manual and Manipulative Therapy*.

[61] Yağci N., Uygur F., Bek N. (2004). Comparison of connective tissue massage and spray-and-stretch technique in the treatment of chronic cervical myofascial pain syndrome. *The Pain Clinic*.

[62] Altan L., Bingöl U., Aykaç M., Yurtkuran M. (2005). Investigation of the effect of GaAs laser therapy on cervical myofascial pain syndrome. *Rheumatology International*.

[63] Chiu T. T. W., Hui-Chan C. W. Y., Cheing G. (2005). A randomized clinical trial of TENS and exercise for patients with chronic neck pain. *Clinical Rehabilitation*.

[64] Chow R. T., Heller G. Z., Barnsley L. (2006). The effect of 300 mW, 830 nm laser on chronic neck pain: a double-blind, randomized, placebo-controlled study. *Pain*.

[65] Dundar U., Evcik D., Samli F., Pusak H., Kavuncu V. (2007). The effect of gallium arsenide aluminum laser therapy in the management of cervical myofascial pain syndrome: a double blind, placebo-controlled study. *Clinical Rheumatology*.

[66] Esenyel M., Caglar N., Aldemir T. (2000). Treatment of myofascial pain. *American Journal of Physical Medicine and Rehabilitation*.

[67] Gam A. N., Warming S., Larsen L. H. (1998). Treatment of myofascial trigger-points with ultrasound combined with massage and exercise—a randomised controlled trial. *Pain*.

[68] Gur A., Sarac A. J., Cevik R., Altindag O., Sarac S. (2004). Efficacy of 904 nm Gallium Arsenide low level laser therapy in the management of chronic myofascial pain in the neck: a double-blind and randomize-controlled trial. *Lasers in Surgery and Medicine*.

[69] Özdemir F., Birtane M., Kokino S. (2001). The clinical efficacy of low-power laser therapy on pain and function in cervical osteoarthritis. *Clinical Rheumatology*.

[70] Seidel U., Uhlemann C. (2002). A randomised controlled double-blind trial comparing dosed lasertherapy on acupuncture points and acupuncture for chronic cervical syndrome. *Deutsche Zeitschrift fur Akupunktur*.

[71] Smania N., Corato E., Fiaschi A., Pietropoli P., Aglioti S. M., Tinazzi M. (2005). Repetitive magnetic stimulation a novel therapeutic approach for myofascial pain syndrome. *Journal of Neurology*.

[72] Thorsen H., Gam A. N., Svensson B. H. (1992). Low level laser therapy for myofascial pain in the neck and shoulder girdle. A double-blind, cross-over study. *Scandinavian Journal of Rheumatology*.

[73] Bland J. M., Altman D. G. (2011). Comparisons within randomised groups can be very misleading. *British Medical Journal*.

[74] Boutron I., Dutton S., Ravaud P., Altman D. G. (2010). Reporting and interpretation of randomized controlled trials with statistically nonsignificant results for primary outcomes. *The Journal of the American Medical Association*.

[75] Higgins J. P., Altman D. G., Gotzsche P. C. (2011). The Cochrane Collaboration's tool for assessing risk of bias in randomised trials. *British Medical Journal*.

[76] Moyer C. A. (2009). Between-groups study designs demand between-groups analyses: a response to hernandez-reif, shor-posner, baez, soto, mendoza, castillo, quintero, perez, and zhang. *Evidence-based Complementary and Alternative Medicine*.

[77] Barton P. M., Hayes K. C. (1996). Neck flexor muscle strength, efficiency, and relaxation times in normal subjects and subjects with unilateral neck pain and headache. *Archives of Physical Medicine and Rehabilitation*.

[78] Falla D., Rainoldi A., Merletti R., Jull G. (2003). Myoelectric manifestations of sternocleidomastoid and anterior scalene muscle fatigue in chronic neck pain patients. *Clinical Neurophysiology*.

[79] Jordan A., Mehlsen J., Ostergaard K. (1997). A comparison of physical characteristics between patients seeking treatment for neck pain and age-matched healthy people. *Journal of Manipulative and Physiological Therapeutics*.

[80] Ylinen J., Salo P., Nykänen M., Kautiainen H., Häkkinen A. (2004). Decreased isometric neck strength in women with chronic neck pain and the repeatability of neck strength measurements. *Archives of Physical Medicine and Rehabilitation*.

[81] Chow R. T., Johnson M. I., Lopes-Martins R. A., Bjordal J. M. (2009). Efficacy of low-level laser therapy in the management of neck pain: a systematic review and meta-analysis of randomised placebo or active-treatment controlled trials. *The Lancet*.

[82] Teasell R. W., McClure J. A., Walton D. (2010). A research synthesis of therapeutic interventions for whiplash-associated disorder (WAD): part 4—noninvasive interventions for chronic WAD. *Pain Research and Management*.

[83] Herbert R. D., Bo K. (2005). Analysis of quality of interventions in systematic reviews. *British Medical Journal*.

